# iCCareD: The Development of an Algorithm to Identify Factors Associated With Distress Among Caregivers of Children and Youth Referred for Mental Health Services

**DOI:** 10.3389/fpsyt.2021.737966

**Published:** 2021-11-18

**Authors:** Shannon L. Stewart, Ashley Toohey, Jeffrey W. Poss

**Affiliations:** ^1^Faculty of Education, Western University, London, ON, Canada; ^2^Faculty of Applied Health Sciences, University of Waterloo, Waterloo, ON, Canada

**Keywords:** caregiver distress, children, children's mental health, interRAI, youth

## Abstract

Caregiver well-being plays an important role in children's development and a number of factors have been found to impact distress levels among caregivers of children and youth referred for mental health services. Further, caregiver distress impacts youth psychopathology, its acuity as well as related mental health interventions. The purpose of this study was to develop and validate an algorithm for identifying caregivers who are at greatest risk of experiencing caregiver distress. This algorithm was derived from, and will be embedded in, existing comprehensive interRAI child and youth instruments. Ontario data based on the interRAI Child and Youth Mental Health assessment instruments (ChYMH and ChYMH-DD) were analyzed to identify predictors of distress among caregivers of children and youth ages 4–18 years. Starting with proactive aggression, the algorithm uses 40 assessment items to assign one of 30 nodes that are grouped into five levels of risk. The interRAI ChYMH Caregiver Distress (iCCareD) algorithm was validated using longitudinal data from mental health agencies across Ontario and was found to be a good predictor among this sample with a c-statistic of 0.71 for predicting new or ongoing caregiver distress and 65% for both sensitivity and specificity using algorithm values of 3 or greater. This algorithm provides an evidence-based decision-support tool embedded within a comprehensive assessment tool that may be used by clinicians to inform their selection of supports and services for families.

## Introduction

The well-being of caregivers has significant implications for the healthy development of children and youth (hereafter referred to as children) (1). Although caregiver distress is expected and typical, it can become problematic if caregivers have difficulty identifying and responding to their children's needs. Research has demonstrated that a number of child, caregiver, and family/environmental characteristics are related to caregivers' stress levels in relation to parenting. Given the important role caregivers play in the development and nurturing of children, understanding the factors that contribute to their distress is crucial.

Much of the research investigating distress in relation to caring for children has focused on child characteristics. Research has consistently shown that children's physical health as well as the behavioral and emotional difficulties they experience, significantly impact their caregivers. With respect to physical health, research has shown that severity and duration of physical illness (2–6) as well as early onset physical health problems of their child (7), are all predictive of greater caregiver distress. That is, caregivers who have children with physical health problems are perceived as more severe or have been ongoing for a longer period of time, experience greater levels of distress in relation to their role as a caregiver. Research findings have also demonstrated that child sleep problems are predictive of greater caregiver distress (5).

In addition to being predicted by physical health factors, there is a well-established relationship between child behavioral and emotional difficulties and caregiver distress (5, 6, 8–11). That is, parents of children who experience greater levels of both externalizing (e.g., aggression) and internalizing (e.g., anxiety, depression) symptomology experience greater levels of caregiver distress. Researchers have found that children with executive functioning problems (i.e., sustaining attention, switching from one task to another, initiating, regulating behavior) have parents who experience more stress (12, 13). Further, those parents whose children were neuro-atypical with co-occurring emotional or behavioral problems reported the highest levels of parenting stress (13). Taken together, these findings highlight the impact of child physical and mental health characteristics on stress levels of their caregivers.

In addition to child-specific characteristics, extant literature examining caregiver distress has underscored the importance of the caregiver themselves as well as the family environment. The relationship between caregiver mental health and distress level is well-established in the literature. Research has shown that caregivers with depression (7) or greater psychiatric co-morbidity (4) experience significantly more caregiver distress. Further, research has shown that caregivers' psychological distress and anxiety are positively correlated with caregivers' feelings of burden and may, in turn, disrupt family well-being (14). In a recent review of the literature, researchers found that parental mental health problems are associated not only with greater caregiver distress, but also lower parenting satisfaction, poorer parent-child relationships, and more frequent use of negative parenting practices (15).

Extant literature has also demonstrated a clear link between financial struggles and caregiver distress. In fact, low family income [e.g., (5, 7)] and other financial stressors have been significantly linked with caregiver distress, accounting for up to 42% of the variance in overall stress reported (8).

In addition to financial difficulties, a perceived lack of social support has also consistently predicted caregiver distress in the literature [e.g., (3, 16)] with stress levels varying by marital quality and perceived level of support (6). Similarly, parents of adolescents with executive functioning issues report greater feelings of isolation, more conflict with their partner, and greater feels of guilt and incompetence (11), all of which are related to their overall caregiver distress levels. Research has also demonstrated that parents with intellectual disabilities are at greater risk of experiencing financial disadvantages and social isolation [e.g., (17, 18)]. Further, research has demonstrated that a lack of social support is related to increased parenting stress for mothers with intellectual disabilities as well as increased behavioral problems for their school-aged children (19). These results highlight that there are a number of caregiver and family environment characteristics that impact caregiver distress and underscore possible interactions of these factors can have an even greater impact on the distress experienced by caregivers.

Though each of these characteristics can individually be associated with child maladjustment, there appears to also be a cumulative effect of caregiver distress and family instability on child well-being (20). Further, the transactional model of development highlights that child and caregiver characteristics impact one another (21). This theoretical framework is supported by research underscoring the dynamic interactions among child, caregiver, and family environment factors that impact caregiver distress. Notably, extant literature has demonstrated that caregiver distress impacts family functioning, child psychopathology, mental health interventions for children [e.g., (1)], and risk of adverse childhood experiences [ACEs; (22)].

Findings from a recent review of parenting stress measures outline that there are a number of well-studied measures of parenting stress which have the ability to assess parenting stress accurately and reliably (1). Instruments have been utilized to assess the burden and strain of parenting children and youth presenting with mental health issues (1). For example, the Parenting Stress Scale (23) was designed to capture the parent's perception of the parental role rather than sources of stress (24). Similarly, the Caregiver Strain Questionnaire (25) has been utilized to assess the burden of caring for children and youth with serious emotional and behavioral issues in order to support clinical interventions and treatment outcomes. Also, the Parenting Stress Index (26) is an inventory that evaluates levels of stress within the parenting role that includes life stress, child characteristics as well as parenting factors. While the ability for identified measures to assess parenting distress accurately and reliably has been strong (1), to our knowledge, no previous research has developed a unique algorithm to predict on-going or future caregiver distress utilizing a comprehensive assessment system. These existing measures are stand-alone and are meant to be used in conjunction with a number of other child-focused assessment measures in order to gain an understanding of child and caregiver functioning. Further, none of these measures include psychometrics with respect to discriminative validity (1), underscoring the need to understand the combination of factors that identify high risk families earlier to provide appropriate and necessary supports and prevent possible ACEs. Given that caregiver distress is relevant to the development, continuation and treatment of childhood mental health issues, the ability to predict which caregivers are at greater risk of experiencing distress would be helpful for clinicians working with children and their families for prevention and early intervention efforts.

The purpose of this study is to develop a new decision-support algorithm embedded within existing assessment tools (the interRAI Child and Youth Mental Health suite). Developing such an algorithm will assist clinicians who are already administering the ChYMH or ChYMH-DD with identifying caregivers at greatest risk of caregiving-related distress. Based on the literature extant, it was anticipated that certain child, parent and environmental factors would be associated with increased levels of caregiver distress. With respect to child behaviors, it was anticipated that sleep problems, emotion dysregulation, aggression, and self-harm behaviors would increase levels of parental distress. Furthermore, it was anticipated that parents who are struggling with family issues, major life stressors, marital problems, domestic violence, as well as difficulties related to their own health and well-being would be at heightened risk for caregiver distress.

## Materials and Methods

### Sample

Participants in this study consisted of children aged 4–18 years who received services from over 50 mental health agencies across Ontario as part of standard of care. Assessments completed between January 2015 and February 2020 were abstracted to an analytic dataset, removing any personal identifiers. These children were referred to the agencies through family physicians, pediatricians, school personnel, parents, or other allied health professionals. After removing records where the client age was outside of the range of 4–18 years, as well as assessments where there was evidence that a parent or other primary caregiver was not present in the child's life, our sample consisted of 30, 210 eligible assessment records. In order to evaluate the data longitudinally, consecutive pairs of assessments for clients were then selected where the two assessments fell between 30 and 365 days apart, and where the first assessment of the pair was not designated as a discharge assessment. If more than one such pair could be constructed for a client, the first one was selected, resulting in 7,182 pairs for analysis. Given that assessment responses are entered by clinicians into computer forms that require all items to be completed to have responses that are of the proper form, no missing data are encountered. Western University's ethics board granted approval for the secondary analysis of data collected in various agencies throughout the Province of Ontario (REB #106415).

### Measures

The Child and Youth Mental Health [ChYMH; (27)] and Child and Youth Mental Health and Developmental Disability [ChYMH-DD; (28)] assessments are comprehensive, clinician-rated standardized, multi-sectoral, and multidisciplinary mental health measures for children and youth, ages 4–18 years. These tools are used as the standard assessment instruments and administered in regular clinical practice in over 50 children's mental health agencies across the province of Ontario. The ChYMH includes over 400 clinical items related to a variety of domains (e.g., mental state indicators, behavior, independence in daily activities, communication, family, and social relations) and the ChYMH-DD includes roughly 65 additional population-specific items. Only the items that are available and identical in both instruments were used to develop the algorithm presented here.

The interRAI ChYMH and ChYMH-DD assessments include items related to the needs, areas of risk, functioning, and strengths of children and youth. These assessments are administered as semi-structured interviews by trained assessors involving the child/youth, guardians, family members as well as collateral contacts (e.g., teachers, therapists). Additionally, information from report cards, academic assessments, medical records, and relevant clinical documents is also reviewed. The tools are intended to support comprehensive care planning, outcome measurement, quality indicators, and case mix classification to estimate relative resource intensity (Stewart and Toohey, under review)^1^.

The result is a valid and reliable set of information that can be used individually for case documentation and to inform program planning as well as collectively for system reporting and secondary research purposes. The interRAI ChYMH and ChYMH-DD are part of an integrated health information system in which psychometrically sound scales and algorithms are embedded within the instrument to support clinical decision making (29–39). A detailed manual supports the instrument and provides coding rules for the items.

Items within the instruments employ specific observation periods in order to provide reliable and valid measures of clinical characteristics that reflect the child's strengths, preferences, and needs. The basic time frame for assessment was set at 3 days unless otherwise indicated. Importantly, some items address the recency and frequency of symptoms prior to and within the last 3 days. There is also an option to indicate that symptoms are present but not exhibited within the last 3 days. Responses for items are constrained, almost always binary or ordinal, and are well-suited to completion using a computer entry format. These tools include a number of scales in order to support care planning.

### Dependent Variable

The target variable of the modeling was the item “parent/primary caregiver expresses feelings of distress, anger, or depression,” coded as no or yes, for the assessment completed on the follow-up assessment. Since the baseline assessment also recorded caregiver distress, the result was to model two variations on this outcome. The first, where caregiver distress is absent at baseline, makes the target outcome that of a newly developed state of caregiver distress. The second, where caregiver distress is present at baseline, makes it one of ongoing distress. By effectively combining this, the model explores factors related to new caregiver distress that might emerge, or if currently present, whether it fails to improve. This so-called “double-barrel” perspective has been applied elsewhere, for example in quality indicator formulation (40).

### Independent Variables

Explanatory items came from the child's baseline assessment. All individual clinical items were collected, gathering information about the child's well-being and development across a variety of domains, including information related to parenting discipline, monitoring, distress in relation to caregiving, and whether the child's parent has a mental health, developmental, or substance use issue. In addition to individual items, a number of computed scales were used as predictors. These included:

- Proactive aggression (7 items: stealing, elopement attempts/threats, bullying peers, preoccupation with violence, violence to others, intimidation of others or threatened violence, violent ideation; 30)
- Reactive aggression [5 items: impulsivity, physical abuse, outbursts of anger, defiant behavior, argumentativeness; (29)]
- Parenting strengths scale (6 items, 0–12, higher values indicate greater parenting strength)
- Disruptive/aggressive behavior scale [5 items, 0–20: verbal abuse, physical abuse, socially inappropriate/disruptive, destructive behavior toward property, outbursts of anger; (31)]
- ChYMH aggressive behavior scale [4 items, 0–12: verbal abuse, physical abuse, socially inappropriate/disruptive, resists care; (2)]
- Family functioning composite score (modified)—(4 items, 0–4: strong/supporting relationship with family, family persistently hostile/critical of child, parent has developmental/mental health/addiction issues, sibling has developmental/mental health/addiction issues).

### Procedure

Seventy-five percent of assessments were randomly assigned to a derivation subset and the remaining 25% were assigned for validation. The analytic method used was an interactive decision tree tool supported by SAS Enterprise Miner. A decision tree starts with all observations that are then sub-divided at branch points into two or more groups based on the value of a clinical item, with these groups subsequently sub-divided using other clinical items until a terminal node is reached. Decision trees have the strength of identifying natural interactions of clinical items (41), such that an item may have a strong association with the outcome in one subset of the population, while different items are important among another. With this tool, the analyst is presented with a list of clinical items and their associated strength of association with the outcome (caregiver distress). A large number of possible variations of trees are possible, with the initial split being particularly important. Several options for the initial split were explored, including characteristics of the family, age of the child/youth, and a summary measure of child/youth aggression. Construction of a candidate tree continues until the number of remaining cases becomes too small, or there are no clinical items that reach a statistical threshold for a split.

The terminal nodes from the completed tree are subsequently gathered to form a number of discrete groups. A design goal was to have a compact scale of five levels. K-means clustering was used to inform the grouping of the decision tree terminal nodes into the five groups. Five candidate trees representing different first splits and designs were fully developed after considerable exploration, and it was felt that additional options would not yield better candidates from a clinical or performance perspective. These five options were assessed, guided by the scale distribution, mean values of the outcome at each level, and goodness of fit using logistic regression. Feedback from content experts was sought leading to additional refinement of the best candidate until a final tree model was selected. Additional subgroup testing by age groups and by sex was conducted.

## Results

Table 1 outlines characteristics of the sample at the baseline assessment. Statistical tests for Table 1 were chi-squares, except for age which is a student *t*-test. Please note that multiple test correction was not completed for the data presented in Table 1. Group comparisons presented in Table 1 highlight that in families where caregivers report feelings of distress, there are poorer interpersonal relationships, higher rates of caregiver mental health/developmental issues, and higher rates of caregivers reporting that they are unwilling to continue caring for the child. Further, in these families, it is more likely that caregivers have made economic trade-offs, have experienced a major life stressor in the last 3 months, and that the physical home environment is cause for concern.

**Table 1:** Sample characteristics at baseline.

***N* (% of column)**	**Caregiver distress**		**All (*N* = 7,182)**	***p*-value**
	**No (*N* = 4,363)**	**Yes (*N* = 2,819)**		
Age: mean (SD)	11.5 (3.5)	11.8 (3.4)	11.6 (3.5)	0.002
Female sex	1,927 (44.2%)	1,143 (40.6%)	3,070 (42.8%)	0.003
Legal guardianship: both parents	2,785 (63.8%)	1,561 (55.4%)	4,346 (60.5%)	<0.0001
Mother only	1,079 (24.7%)	931 (33.0%)	2,010 (28.0%)	<0.0001
Father only	171 (3.9%)	88 (3.1%)	259 (3.6%)	0.077
Other relative or non-relative	196 (4.5%)	145 (5.1%)	341 (4.8%)	0.205
Other (child protection, public guardian, youth responsible for self)	132 (3.0%)	94 (3.3%)	226 (3.2%)	0.464
Current custody dispute	203 (4.7%)	146 (5.2%)	349 (4.9%)	0.311
Marital status of parents: married	1,977 (45.3%)	1,086 (38.5%)	3,063 (42.7%)	<0.0001
Separated or divorced	1,187 (27.2%)	830 (29.4%)	2,017 (28.1%)	0.039
Never married	804 (18.4%)	632 (22.4%)	1,436 (20.0%)	<0.0001
Other	395 (9.1%)	271 (9.6%)	666 (9.3%)	0.424
Any history of foster placement	447 (10.3%)	419 (14.9%)	866 (12.1%)	<0.0001
Lives with parent or primary caregiver	4,090 (93.8%)	2,564 (91.1%)	6,654 (92.7%)	<0.0001
Strong, supportive relationship with family	3,979 (91.2%)	2,178 (77.3%)	6,157 (85.7%)	<0.0001
Family engaged in/supportive of treatment	4,080 (93.5%)	2,642 (93.7%)	6,722 (93.6%)	0.726
Conflict, repeated criticism of family	899 (20.6%)	1,446 (51.3%)	2,345 (32.7%)	<0.0001
Conflict, repeated criticism of close friends	394 (9.0%)	505 (17.9%)	899 (12.5%)	<0.0001
Family hostile/critical of child/youth	373 (8.6%)	778 (27.6%)	1,151 (16.0%)	<0.0001
Friends hostile/critical of child/youth	230 (5.3%)	330 (11.7%)	560 (7.8%)	<0.0001
Conflict with peers (excluding close friends)	774 (17.7%)	835 (29.6%)	1,609 (22.4%)	<0.0001
Conflict/criticism between parents/caregivers	619 (14.2%)	957 (34.0%)	1,576 (21.9%)	<0.0001
Family members feeling overwhelmed	1,189 (27.3%)	2,237 (79.4%)	3,426 (47.7%)	<0.0001
Parent/caregiver overprotective	374 (8.6%)	498 (17.7%)	872 (12.1%)	<0.0001
Parent/caregiver is intrusive	140 (3.2%)	275 (9.8%)	415 (5.8%)	<0.0001
Parent/caregiver has mental health or developmental issue	1,411 (32.3%)	1,627 (57.7%)	3,038 (42.3%)	<0.0001
Parent/caregiver unable/unwilling to continue care	44 (1.0%)	291 (10.3%)	335 (4.7%)	<0.0001
Finances: made economic trade-offs	74 (1.7%)	192 (6.8%)	266 (3.7%)	<0.0001
Parent/caregiver major life stressor last 90 days	751 (17.2%)	1,133 (40.2%)	1,884 (26.2%)	<0.0001
Home environment, any of: disrepair, squalor, heating/cooling	67 (1.5%)	149 (5.3%)	216 (3.0%)	<0.0001
inadequate, lack of personal safety, access to home, or rooms in home				

**Figure 1:**
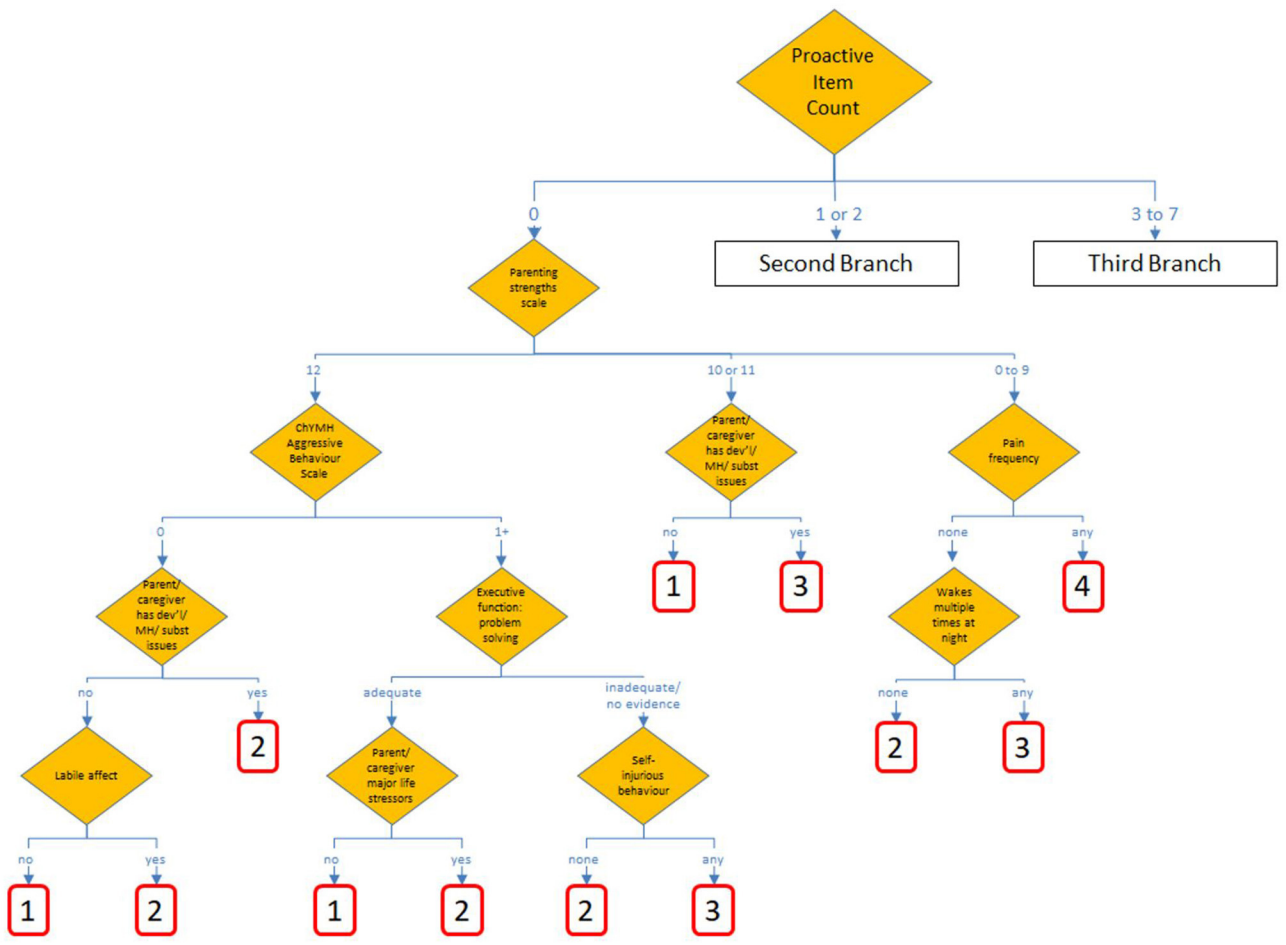
First branch of ChYMH Caregiver Distress Tree Algorithm.

**Figure 2:**
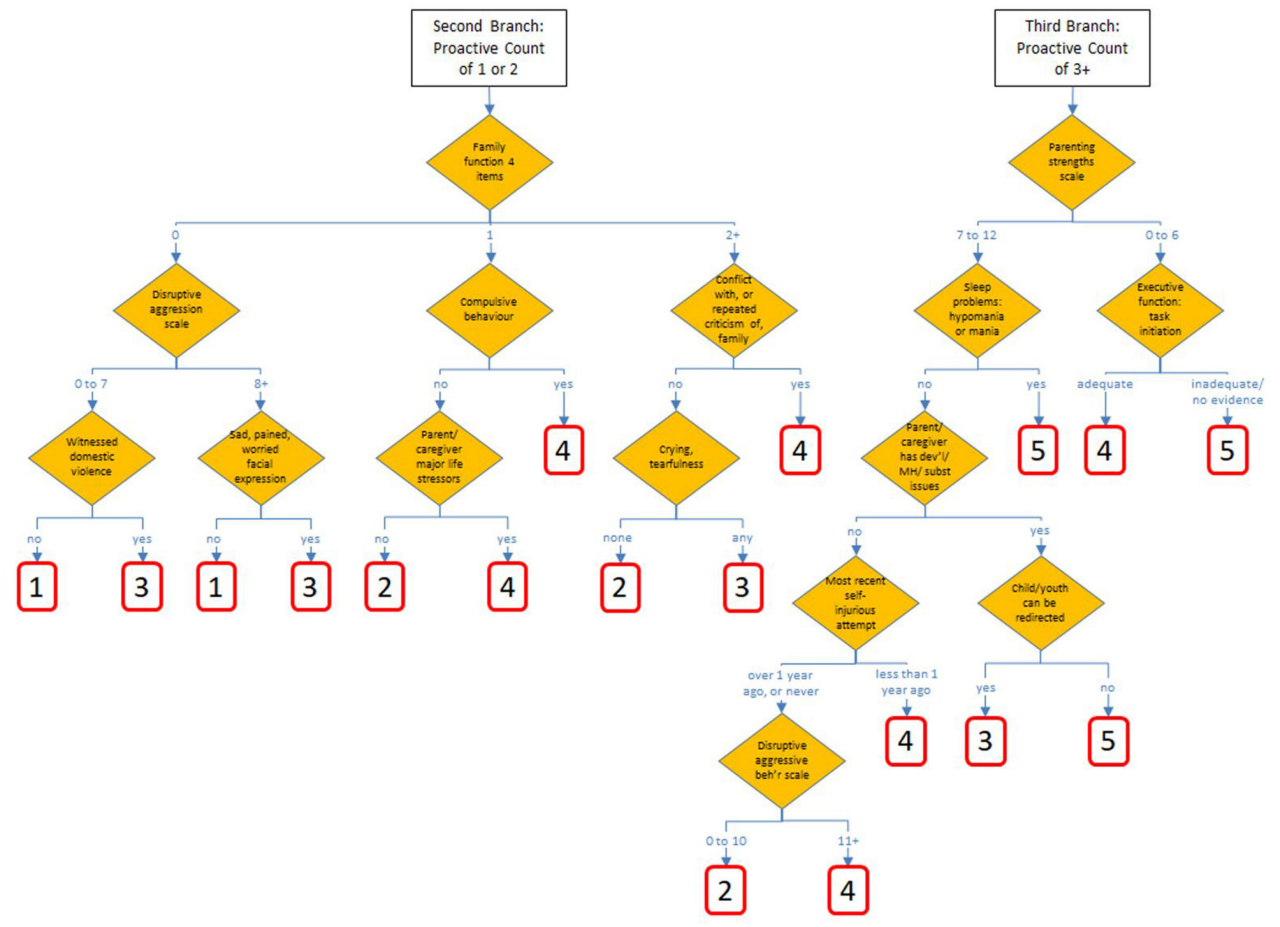
Second and third branches of ChYMH Caregiver Distress Tree Algorithm.

The candidate tree that used proactive aggression as the initial split produced a tree with better performance than others considered, including those split initially by age, sex, other aggregations of parent/caregiver strengths/function or child/youth behavior. Figures 1, 2 show the chosen tree diagram for the assignment of the 30 nodes, and their subsequent assignment to a scale value. The first split is into three groups: those cases with none of 7 proactive aggressive items, those with 1 or 2, and those with 3 or more.

In the chosen tree, all nodes are conditioned on characteristics of the child/youth as the first split, and by characteristics of the family, parent, or primary caregiver which is used as the second split in each of the three branches. There are 40 different items drawn from these eight sections of the assessment: mental state indicators, harm to self or others, behavior, strengths and resilience, executive functioning, health conditions, family/social relations, and stress/trauma.

Overall, 28.1% of the parent/primary caregivers were recorded as having new or ongoing distress, 13.4% new distress (among those without distress at baseline), and 48.7% had ongoing distress (among those with distress at baseline). The distribution and results of the five-point scale are shown in Table 2. Across the range of the scale, there is an 8-fold difference in rates of new or ongoing distress, 10-fold difference for new distress, and a 2.3-fold difference for ongoing distress.

**Table 2:** ChYMH Caregiver Distress Algorithm results.

**Algorithm value**	***N* (%)**	**New or ongoing distress**	New distress^*^	Ongoing distress^**^
1	1,869 (26%)	8.2%	3.8%	33.7%
2	2,209 (31%)	21.3%	9.7%	43.0%
3	1,372 (19%)	35.8%	14.6%	57.7%
4	1,229 (17%)	46.5%	23.9%	60.3%
5	503 (7%)	66.6%	38.1%	77.0%
All	7,182 (100%)	28.1%	13.4%	48.7%

* *Among those without distress at time 1, n = 4,363*.

** *Among those with distress at time 1, n = 2,819*.

Goodness of fit, the area under the curve or C-statistic from logistic regression models, is presented in Table 3. C-statistic values of 0.5 represent performance no better than chance, while values of 1.0 represent performance where the algorithm predicts all observations perfectly. Considering the dependent variable that was modeled, new or ongoing distress, the reserved validation sample is somewhat lower, which is expected for this kind of modeling where there is a tendency to over-fit the derivation data, compared to the validation data. The difference in fit (0.740 vs. 0.706) here is to be noted, and with future data the degree of over-fitting could be gauged more precisely. Fit was slightly better for female clients than for males, and slightly poorer for the youngest clients. Goodness of fit was not as strong for the related, but not directly modeled, outcomes of new distress as well as ongoing distress. Values for sensitivity and specific, using algorithm values of 3 or greater, were found to be 69 and 67% in the derivation sample, and 65 and 65% in the validation sample.

**Table 3:** ChYMH Caregiver Distress Algorithm goodness of Fit C-statistics^*^.

	**New or ongoing distress**	**New distress**	**Ongoing distress**
Derivation	0.740	0.701	0.643
Validation	0.706	0.648	0.617
All	0.731	0.686	0.636
Males	0.721	0.682	0.630
Females	0.743	0.692	0.647
Age 4–7 years	0.699	0.585	0.623
Age 8–11 years	0.754	0.704	0.656
Age 12–18 years	0.730	0.703	0.628

* *Area under the receiver-operator characteristic curve, or AUC*.

There are no instances of reversal (i.e., where a higher scale value results in a lower proportion of the outcome) for the dependent variable among the age and the sex stratifications. In addition, there are no reversals observed for the outcomes of new distress and of ongoing distress. Fewer than 2.5% of the assessment pairs came from the Development Disabilities version of the ChYMH, too few to conduct sub-group analyses specific to these cases.

## Discussion

Our results indicate that nearly half of the caregivers who were experiencing distress at baseline continued to experience distress at time two. Further, 13.4% of caregivers who were not experiencing distress initially were experiencing distress at the follow-up assessment. Given the negative consequences associated with caregiver distress for both caregiver and child well-being, these findings highlight the need for clinicians working with children and their families to evaluate and care plan for caregiver distress. Overall, our model highlights that greater proactive aggressive behavior is related to increased likelihood of caregiver distress. This result is consistent with the well-established relationship in the literature between externalizing behaviors and caregiver distress [e.g., (15, 42)]. These results highlight the importance of checking in with caregivers as to their well-being when children present with difficulties related to stealing, elopement, bullying, and violence.

Of note, with respect to new or ongoing distress, our model was a slightly better fit for child/caregiver dyads where the child was 8 years of age or older than for children 4–7 years old. This finding is not surprising given the extant literature highlighting that age of onset of mental health issues is negatively related to time to initial professional help-seeking [e.g., (43, 44)]. Moreover, results from a large-scale study found that the average delay among those with anxiety disorders who eventually made treatment contact was 6–8 years and 9–23 years for mood disorders (44). Taken together with the fact that the age of onset for anxiety disorders tends to be younger than that for mood, behavioral, and substance use disorders (45), it is possible that younger children (and their caregivers) are more unpredictable in their future progression with mental health services than older children and adolescents.

Consistent with previous literature, characteristics of both the child and the caregiver/caregiving environment both predicted caregiver distress. That is, our results demonstrate that among a large sample of children referred for mental health services, the model that best fit the data to predict caregiver distress included aggressive behavior of the child at the first level, followed by parenting strengths or family functioning at the second level. Our algorithm demonstrates that parents/caregivers who did not communicate effectively with their child, had difficulty assisting their child with emotion regulation and who did not demonstrate warmth and support to their child were less likely to utilize appropriate disciplinary practices and were more likely to experience increased distress. These findings are consistent with research demonstrating that caregiver distress is related to both parenting competence and parental responsiveness (46). Previous research has shown that parents who report elevated stress and perceive their child as being “difficult,” typically lack warmth and responsiveness in their interactions with their child, have developmentally inappropriate expectations for their child's behavior, and use inconsistent discipline practices [e.g., (47)]. The nature of the relationship between caregivers, and children and the level of support caregivers provide their children with, are clear indicators of the level of distress these caregivers are experiencing. Our findings also echo previous research findings suggesting that negative parent/child interactions (i.e., lack of emotional warmth, feelings of rejection) as well as experiencing major life stressors are significantly related to parenting stress (48). Further, families in which there are high levels of involvement and cohesion report lower levels of parenting stress (49). Given the impact of caregivers' perceptions of their own efficacy and enjoyment of parenting on their levels of distress, it is not surprising that those caregivers who experience negative relationships with their children, and who recently experienced a major stressful life event, would experience greater caregiver distress. Further, research has demonstrated that controlling and hostile parenting and interaction styles (50), and poor family social cohesion (51, 52) have all been associated with child internalizing disorders. Findings in our model underscore the important role that family and parent/child dynamics, as well as both child and caregiver well-being play with regards to caregiver distress.

With respect to characteristics of the child, our findings indicate that a child's high pain frequency predicted increased likelihood of caregiver distress, a finding consistent with previous research highlighting that severity and duration of physical health problems among children significantly predicts greater caregiver distress [e.g., (6, 9)]. Similar to other literature extant, child sleep problems were also related to increased risk of caregiver distress (9) as was compulsive child behaviors [e.g., (12, 13)]. It was not surprising that executive functioning difficulties in children were associated with increased caregiver distress given that such difficulties require ongoing supports from caregivers to remain on-task, resulting in the need for constant reminders and prompts for redirection (17). Finally, with respect to child characteristics, self-injurious attempts, and disruptive/aggressive behavior were also associated with caregiver distress, a consistent finding throughout the literature (53–56).

Consistent with previous literature [e.g., (8, 11)], for those children who demonstrated the most proactive aggression, in conjunction with those caregivers who exhibited developmental, mental health, or substance use issues, high levels of distress were noted. Similarly, parents with children with intellectual disabilities were more likely to experience financial difficulties and lack social support (22), both of which are related to increased caregiver distress.

Research has demonstrated the negative consequences of caregiver distress for children's development. More specifically, higher caregiver distress has been related to internalizing difficulties among children (57), lack of social competence, as well as externalizing behaviors (58). Further, research has also demonstrated that reducing parenting stress is related to less coercive, harsh parenting practices (59), thereby reducing the possible adverse consequences that elevated caregiver distress has on children and families. Identifying factors associated with caregiver distress can help facilitate the implementation of strategies to target those factors, thereby potentially reducing a variety of negative sequelae including risk of ACEs (22).

### Use and Utility

Based on the findings, the ChYMH Caregiver Distress Algorithm is an empirically based decision-support tool that may be used to identify those who are at greater risk of experiencing caregiver distress. Service providers who have completed the interRAI ChYMH or ChYMH-DD assessment can obtain the iCCareD scores automatically from the software in which the algorithm is embedded, and these results can then provide insight as to the service needs for the child and family. It should be noted however, that the intent is not to use the ChYMH Caregiver Distress algorithm as an automated decision-making system. Rather, the iCCareD score, along with other information obtained during the assessment process, should be used to assist the clinical team when determining the level and kinds of support needed by the family. That is, if the iCCareD score is in the upper range, the clinical team could consider collecting additional information about the caregiver's well-being in order to further support care planning for the caregiver specifically (1), or provide referrals for the caregiver to receive additional individual supports. The clinical team should use their professional and clinical judgement to determine whether the score accurately reflects those with distress that may be persistent, or those without evident distress who may be at greater risk of developing it. If a score is in the upper range, it is recommended that the clinical team consider the caregiver to be at high need for support in order to reduce their distress level or to reduce the likelihood that the caregiver will develop distress due to their heightened risk level. If the score is in the lower range, it is recommended that further discussion occur to determine whether the level of distress is appropriate. In all situations, the child's caregiver should be involved in the decision-making process and consider their preferences as well as their strengths and needs (60). For example, a parent with a very high score on the iCCareD algorithm may not require intensive services because adequate supports and other family members are able to address their current needs.

In addition to being used for individualized caregiver support decisions, the iCCareD algorithm can also provide standardized, comprehensive data across agencies, allowing for the identification of needs across the system. Similar to other interRAI algorithms, populations can be stratified according to the Caregiver Distress levels and then be used to compare the performance of mental health agencies with respect to outcomes of care within the Caregiver Distress levels. The main benefit of implementing the iCCareD algorithm is that those individuals who are at greatest risk of experiencing distress will be identified more quickly and accurately and will be provided with more focused services and supports. At the same time, this is not meant to limit the supports provided for caregivers who are at the lower level.

Caregiver distress levels can also be evaluated at the regional, organization, national, and international levels to develop a benchmarking system (61) that can be used to identify jurisdictions where caregiver distress levels are higher than in other regions. This would allow comparisons of similar populations and can be used to inform policy development and planning. Additionally, caregiver distress levels at intake can be used to examine variations across regions with respect to how services are used based on level of need.

While this study has numerous strengths, including its relatively large sample size and longitudinal approach, the findings of this study are not without limitations. First, the results reported herein may not be generalizable to a community-based sample because the sample consisted of parents/caregivers of children who were accessing mental health services. Second, items chosen in the algorithm were selected based on both clinical relevance and statistical power, but do not represent all possible factors that may drive caregiver distress.

## Data Availability Statement

The datasets presented in this article are not readily available due to the highly sensitive and confidential nature of the data, as well as the ethical requirements required for use, data will not be made freely available. Moreover, participating mental health agencies required that data not be made freely accessible. Requests to access the datasets should be directed to Shannon L. Stewart, sstewa24@uwo.ca.

## Ethics Statement

Ethics approval for secondary data analyses of interRAI data gathered by other organizations was obtained through Western University REB (106415).

## Author Contributions

The procedure and execution of the study was completed by SS. Data analysis was performed by JP in consultation with SS. The first draft of the manuscript was written by AT. SS and AT provided revised versions the manuscript. All authors contributed to the conception of this study, read, and approved the final manuscript.

## Funding

This work was partially supported by the Public Health Agency of Canada under grant #1617-HQ-000050 and the Canadian Institutes of Health Research under grant #412154.

## Conflict of Interest

The authors declare that the research was conducted in the absence of any commercial or financial relationships that could be construed as a potential conflict of interest.

## Publisher's Note

All claims expressed in this article are solely those of the authors and do not necessarily represent those of their affiliated organizations, or those of the publisher, the editors and the reviewers. Any product that may be evaluated in this article, or claim that may be made by its manufacturer, is not guaranteed or endorsed by the publisher.

## Footnotes

^1^Stewart SL, Toohey A. Screening assessment of mental health problems in students: using an integrated assessment-to-intervention approach to enhance integrated care. In: Jacques JS, Shaw S, Domene J, McMorris C, editors. *Mental Health Assessment, Prevention, and Intervention*: *Promoting Child and Youth Well-Being to be Published in the Springer Series on Human Exceptionality* (under review).
