# Supplementary material for: iCCareD: The Development of an Algorithm to Identify Factors Associated With Distress Among Caregivers of Children and Youth Referred for Mental Health Services
Source: Front Psychiatry. 2021 Nov 18;12:737966. doi: 10.3389/fpsyt.2021.737966 (PMC8637612; doi:10.3389/fpsyt.2021.737966)
Supplement: Supplementary file 1 [file Data_Sheet_1.docx]

Supplementary Material

# Supplementary Tables

**Supplementary Table 1**

*ChYMH Caregiver Distress Algorithm Results: Males Only*

| Scale value | *N* (%) | New or Ongoing Distress | New Distress* | Ongoing Distress** |
| --- | --- | --- | --- | --- |
| 1 | 1,015 (25%) | 8.7% | 4.1% | 32.3% |
| 2 | 1,261 (31%) | 21.5% | 10.8% | 43.6% |
| 3 | 790 (19%) | 36.5% | 15.2% | 56.2% |
| 4 | 724 (18%) | 44.3% | 21.8% | 58.1% |
| 5 | 322 (8%) | 64.6% | 31.7% | 75.3% |

* Among those without distress at time 1, n=2,436

** Among those with distress at time 1, n=1,676

**Supplementary Table 2**

*ChYMH Caregiver Distress Algorithm Results: Females Only*

| Scale value | *N* (%) | New or Ongoing Distress | New Distress* | Ongoing Distress** |
| --- | --- | --- | --- | --- |
| 1 | 854 (28%) | 7.7% | 3.7% | 35.9% |
| 2 | 948 (31%) | 21.0% | 11.0% | 42.2% |
| 3 | 582 (19%) | 34.9% | 13.7% | 59.9% |
| 4 | 505 (16%) | 49.5% | 24.2% | 63.3% |
| 5 | 181 (6%) | 70.2% | 36.6% | 80.0% |

* Among those without distress at time 1, n=1927

** Among those with distress at time 1, n=1143

**Supplementary Table 3**

*ChYMH Caregiver Distress Algorithm Results: Children 7 years of Age and Younger*

| Scale value | *N* (%) | New or Ongoing Distress | New Distress* | Ongoing Distress** |
| --- | --- | --- | --- | --- |
| 1 | 286 (28%) | 10.1% | 7.6% | 22.5% |
| 2 | 326 (32%) | 20.6% | 10.7% | 42.6% |
| 3 | 232 (23%) | 31.0% | 13.3% | 52.9% |
| 4 | 139 (14%) | 33.1% | 15.9% | 47.4% |
| 5 | 44 (4%) | 61.4% | 28.6% | 67.6% |

* Among those without distress at time 1, n=660

** Among those with distress at time 1, n=367

**Supplementary Table 4**

*ChYMH Caregiver Distress Algorithm Results: Children Ages 8 to 11 years*

| Scale value | *N* (%) | New or Ongoing Distress | New Distress* | Ongoing Distress** |
| --- | --- | --- | --- | --- |
| 1 | 634 (26%) | 6.0% | 2.9% | 28.2% |
| 2 | 723 (30%) | 19.4% | 10.7% | 39.8% |
| 3 | 474 (20%) | 35.2% | 12.3% | 57.7% |
| 4 | 384 (16%) | 44.5% | 22.1% | 57.4% |
| 5 | 179 (7%) | 70.4% | 36.6% | 80.4% |

* Among those without distress at time 1, n=1,479

** Among those with distress at time 1, n=915

**Supplementary Table 5**

*ChYMH Caregiver Distress Algorithm Results: Children Ages 12 to 18 years*

| Scale value | *N* (%) | New or Ongoing Distress | New Distress* | Ongoing Distress** |
| --- | --- | --- | --- | --- |
| 1 | 949 (25%) | 9.2% | 3.6% | 10.6% |
| 2 | 1,160 (31%) | 22.7% | 11.1% | 44.8% |
| 3 | 666 (18%) | 37.8% | 16.5% | 59.2% |
| 4 | 706 (19%) | 50.2% | 24.8% | 64.0% |
| 5 | 280 (7%) | 65.0% | 31.9% | 76.4% |

* Among those without distress at time 1, n=2,224

** Among those with distress at time 1, n=1,537
